# Indometacin induced corneal perforation in a patient with rheumatoid arthritis

**DOI:** 10.11604/pamj.2018.29.16.9649

**Published:** 2018-01-08

**Authors:** Fouad Chraibi, Idriss Andaloussi Benatiya

**Affiliations:** 1 University Allal Ben Abdellah, University Hospital Hassan II, Fez, Morocco

**Keywords:** Corneal perforation, indometacin, rheumatoid arthritis

## Image in medicine

Severe corneal complications associated with the use of anti inflammatory drugs (NSAID) eye drops are known. We report the case of a patient of 57 years treated for rheumatoid arthritis with a central corneal perforation of the right eye following the use of indomethacin eye drops in the postoperative period following cataract surgery. The image shows a paracentral corneal perforation with iris prolaps. The course was favorable with improvement in visual acuity and complete healing of the corneal perforation after stoping indometacin and using of bandage contact lens. The anti inflammatory drugs should be avoided in cases of rheumatoid arthritis because they increase the risk of perforation. Indomethacin, like other molecules in the same therapeutic class should be used with caution in patients with general risk factors of suffering from ocular surface.

**Figure 1:**
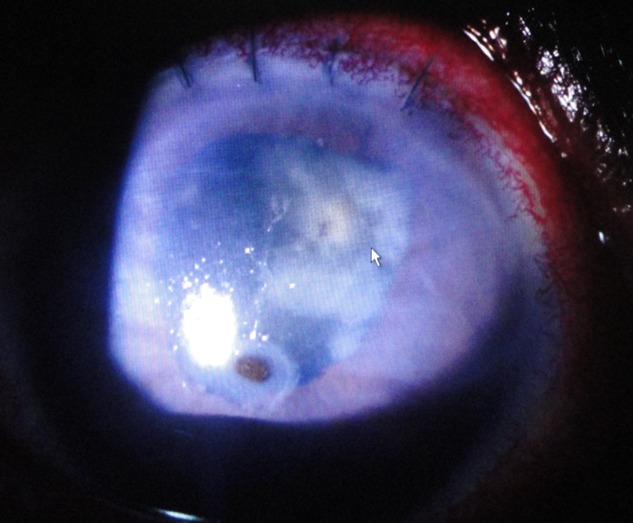
Paracentral corneal perforation

